# College Students’ Views on Functional, Interactive and Critical Nutrition Literacy: A Qualitative Study

**DOI:** 10.3390/ijerph18031124

**Published:** 2021-01-27

**Authors:** Jade McNamara, Noereem Z. Mena, Leigh Neptune, Kayla Parsons

**Affiliations:** 1 School of Food and Agriculture, University of Maine Orono, Orono, ME 04469, USA; leigh.neptune@maine.edu (L.N.); kayla.l.parsons@maine.edu (K.P.); 2 Department of Food Science and Human Nutrition, Colorado State University, Fort Collins, CO 80523, USA; mnoereem@gmail.com

**Keywords:** nutrition literacy, eating behavior, young adults

## Abstract

This research aimed to uncover how the nutrition literacy domains (functional, interactive, critical) influence the dietary decisions of young adults in college. For this qualitative study, undergraduate college students aged 18–24 years old (*n* = 24) were recruited to participate in focus groups. The focus group transcripts were independently coded for primary and secondary themes using a grounded theory approach and a basic thematic analysis. Four focus groups with 5–7 participants per group were conducted. The three domains of nutrition literacy emerged in the focus groups with two themes per domain. Themes within functional nutrition literacy included ‘food enhances or inhibits good health’ and ‘components of a healthy diet’; themes within interactive nutrition literacy included ‘navigating the college food environment’ and ‘awareness of food marketing on dietary behavior’; themes within critical nutrition literacy included ‘critical appraisal of nutrition information’ and ‘awareness of societal barriers to good health’. Understanding how the different nutrition literacy domains relate to college students’ food choices can inform future researchers on how to appropriately assess nutrition literacy and design programs aimed at improving dietary behaviors of college students.

## 1. Introduction

Sufficient nutrition-related knowledge, optimal dietary practices and weight management are among the most important modifiable risk factors for health promotion and the prevention of many chronic diseases [1]. According to a 2016 analysis, 73% of adult American men and 63% of adult American women were overweight and over one third were obese [2]. This excess body weight may be attributed, in part, to dietary patterns that are significantly divergent from those recommended in the Dietary Guidelines for Americans (DGA). Research conducted by the Department of Health and Human Services estimated that 75% Americans typically consumed diets low in fruits and vegetables and most consumed excess calories from added sugars and saturated fats [3]. Improving societal dietary habits is an interdisciplinary effort that includes diverse arenas such as cultural competency, resource availability, food-related skills and nutrition education [4].

College undergraduate students who are transitioning from adolescence to young adulthood are at a critical life stage in which they are establishing independence and developing what often become lasting lifestyle behaviors [5]. Weight gain has been found to be common but variable among college undergraduate students with first year college students being identified as a population particularly susceptible to weight gain [6,7]. Those experiencing obesity at this critical time experience a higher incidence of chronic disease later in life [5]. Among the self-identified determinants of college students’ eating behaviors are personal preferences, morals and beliefs, stress, body image, dietary knowledge, convenience, daily routines, social support and environmental factors such as food availability and cost [8].

The determinants of health listed above fit within the socio-ecological model (SEM), which is a framework for health prevention efforts. The SEM encompasses individual, interpersonal, community and societal factors that influence health behaviors [9]. As college students are living and “working” on their college campuses, these four factors play a vital role in health decision-making, which includes healthy eating behaviors. While previous research has investigated the role of the built environment and peer influence on health behaviors, research has not explored how college students use and view nutrition literacy within the context of the SEM when making health decisions.

While there are many factors that influence dietary and lifestyle behaviors, it has been demonstrated that with higher levels of nutrition knowledge and self-efficacy for healthy eating people consume a more healthy diet [9,10]. Nutrition literacy, which encompasses both nutrition knowledge and motivation to eat a healthy diet, has been shown to predict diet quality in differing populations (i.e., people with chronic disease and parents of young children) [11,12]. Velardo [13] described nutrition literacy as a multifaceted concept, outlining three subsets: (1) functional, or the ability to obtain, understand and use factual health information; (2) interactive, or the ability to act effectively to improve health and to communicate, provide and apply relevant health information; (3) critical, or the ability to critically assess health information and advice along with advocating for a more healthful nutrition environment for all [4]. The purpose of this study was to determine if college students describe the nutrition literacy domains (functional, interactive, critical) as they are discussing eating behaviors and, if so, how those domains influence the dietary decisions of young adults in the college undergraduate setting.

## 2. Materials and Methods

### 2.1. Setting, Recruitment and Participants

Data collection took place in February 2019. All methods were approved by the Institutional Review Board at the participating university (protocol code 2018-11-06). Undergraduate students were recruited through class announcements in an introductory level course, posted flyers throughout campus and emails (which were provided by student records) to participate in focus groups to gain feedback on nutrition literacy from a general young adult cohort with potentially less knowledge and exposure to nutrition terminology. Students who were in a health-related major (e.g., nutrition, kinesiology, pre-medical, nursing) were excluded from participating.

### 2.2. Moderator Guide Development

To understand which nutrition literacy domains (functional, interactive and critical) influenced the dietary decisions of college students, a semi-structured focus group guide was developed using the major constructs of nutrition literacy as the theoretical framework [13]. The focus group questions were developed by the lead researcher (J.M.) to prompt discussions among participants related to which and how nutrition literacy domains influenced the dietary decisions of college students. Three content area experts in community nutrition, nutrition education and qualitative data collection methods then reviewed the moderator guide for accuracy and relevance. The final moderator guide had a total of nine questions with three questions per nutrition literacy domain; for example, ‘what comes to mind when you hear the word nutrition?’ and ‘how do you decide if a food product is healthy?’.

### 2.3. Procedure

Four focus groups were conducted by a trained moderator during which undergraduate students were prompted to discuss questions amongst themselves until everyone had the opportunity to participate. After four focus groups were conducted the consensus from the research team was that data had reached saturation and additional focus groups were not necessary. Students received USD 20 for their participation. Prior to conducting each focus group, participants provided informed consent and completed a demographics survey. The demographics questionnaire included items on gender, ethnicity, age and major. An open-ended item was included that asked participants to define in their own words what they thought nutrition literacy was. Students also completed a food questionnaire to assess dietary behaviors [14].

A notetaker was present at each focus group and after each focus group discussion the moderator and notetaker met briefly to discuss key findings and compare findings with the previously conducted focus groups. All focus group discussions were digitally recorded (60 min in length on average). Focus group audio recordings were transcribed verbatim into Microsoft Word (Microsoft Corporation, Redmond, WA, USA, 2015) by two research assistants (K.P. and L.N.) and reviewed for accuracy by a third research assistant (J.M.).

### 2.4. Analysis

Descriptive statistics including frequencies and proportions were calculated for all demographic survey variables using SPSS version 26 (IBM Corp. Released 2019. IBM SPSS Statistics for Windows, Version 26.0. IBM Corp: Armonk, NY, USA). Thematic (content) analysis [15,16] was utilized by N.M. to examine responses and identify themes related to the open-ended item “Define in your own words what you think nutrition literacy is”. Themes identified were reviewed and confirmed with the lead author.

Consistent with the development of the focus group moderator guide, the nutrition literacy domains as described by Velardo [13] were used to develop the codebook. A priori codes were identified to facilitate a systematic coding of the transcripts for emergent themes and categorization of the data into functional, interactive and critical domains. Group coding was then used to train coders and verify a priori codes.

Focus group transcripts were independently coded for primary and secondary themes using a grounded theory approach [17] and a basic thematic (content) analysis [15,16]. First, one research assistant (K.P.) independently coded the transcripts, which were subsequently coded by a second research assistant (L.N.). Emerging and final themes with supporting text segments were then compiled and reviewed for accuracy and any discrepancies were discussed and clarified with the research team. A third independent researcher (N.M.) reviewed all transcripts to verify the findings captured for all a priori and emergent themes within the nutrition literacy domains.

## 3. Results

### 3.1. Participant Demographics

A total of 24 undergraduate students participated in the focus groups. The majority of the students identified as female (70%) and white (77%) with a mean age 20.1 ± 1.5 years old. More than three-quarters of the participants reported natural sciences (57%) and liberal arts and sciences (23%) as their majors. Approximately 57% (*n* = 17) of the participants reported eating fruit just one time a day or less and 76% (*n* = 23) reported eating vegetables two times a day or less. The majority (70%) reported never drinking soda and more than half (57%) reported drinking fruit drinks or sport drinks at least once a week.

### 3.2. Qualitative Results

#### 3.2.1. Defining Nutrition Literacy in Their Own Words

Nutrition literacy was mostly described by students as having a general understanding or knowledge of what foods comprise a healthy diet and how foods consumed impact their bodies and physical health. As one student put it, “The understanding of nutritional facts about food as well as overall health to the body”. Others stated, “Knowing what is in the food we eat and how it interacts with our bodies” and “Knowing the nutritional value of food and the effect it will have on the body”. Few students’ definitions of nutrition literacy included skills to critically assess and apply appropriate information in the context of healthy eating or disease prevention or management. One stated, “Knowing the terminology of nutrition and being able to make choices based on it”. Others stated, “Understanding how to read a nutrition label” and “Being able to discern what is healthy for our bodies and minds”. None of the definitions included aspects of advocating for a more healthful environment for all.

#### 3.2.2. Focus Group Themes

A total of six themes were identified from the focus groups: two per each domain. Themes related to functional nutrition literacy included ‘food enhances or inhibits good health’ and ‘components of a healthy diet’; themes related to interactive nutrition literacy included ‘navigating the college food environment’ and ‘awareness of food marketing on dietary behavior’; themes related to critical nutrition literacy included ‘critical appraisal of nutrition information’ (e.g., scientific studies, books, medical professionals and government websites) for information on nutrition, food production and overall health and wellbeing and ‘awareness of societal barriers to good health’. All themes and additional supporting quotes to those reported in text are available in Table 1.

#### 3.2.3. Functional Nutrition Literacy

With regard to ‘food enhances or inhibits good health’, students reported that they “listen to their bodies” and “just know” which foods are healthy. Students reported relying on how a food or foods made them feel physically to determine if a food was healthy or not and this influenced future food-based decisions. If particular foods had a negative impact on physical wellbeing, students chose to avoid them. The following captures this sentiment expressed by students:

“I guess for me I’d just say, ‘eat stuff that doesn’t make your body feel bad’. I don’t eat as much sugar as I used to. Not so much ‘cause I’m worried about my weight, or my health in the future, but because after I eat a lot of sugar I just feel kind of crappy and I don’t like that.”

“If I eat something, I try to just notice how I feel after I eat it. If I eat something and it makes me feel kind of crappy and heavy and like my head’s in a fog, I just try not to eat it again.”

Knowledge of healthy foods was suggested to be innate by several participants; information one “just knew” as opposed to something learned. As one participant shared:

“I don’t go and research food really and try to figure out what is healthy because I feel like it’s pretty secure in my mind—or I can just like use common sense to figure out if something is going to be healthy or not.”

Another said:

“I feel like, generally it’s pretty common knowledge. [...] I feel like it’s—as far as when it comes to food and stuff there’s not that much left to debate.”

With regard to ‘components of a healthy diet’, students reported using calories as a measure of food intake, nutrition and healthfulness of foods. ‘Calories’ was a common or easily identified nutrition-related term reported by students and used as a tool for determining several eating-based decisions. As one student explained:

“I think people really count their calories, some of them, who don’t really know about nutrition and stuff like carbs or whatever, just say whatever has the least amount of calories should be the healthiest for you.”

Another added:

“I really like to know approximately how many calories I am getting, because if I’m doing weightlifting and stuff like that, I don’t want to be pushing my muscles to the point where they’re not getting enough nutrition or I’m not eating enough. So, I like to get at least a little bit of a general idea of what I’m intaking and what I’m outputting.”

#### 3.2.4. Interactive Nutrition Literacy

With regard to ‘navigating the college food environment’, in general, students described poorer dietary choices and food behaviors within the college environment compared with being outside of the college environment. Furthermore, students also expressed that the college environment made it challenging to eat healthfully. College or social gatherings were described to primarily center around less healthful foods and alcohol subsequently leading to excess calorie intake. Inconsistent or busy schedules caused disruptions in eating patterns and led to a prioritization of convivence foods due to limited time and resources. These behaviors were recognized to have negative impacts on long-term health, for example:

“The whole communal college student bonding rituals, which almost always involve either drinking, which entails a lot of calories, or eating, which also entails a lot of calories… you can cut yourself out of that particular social circle or you can just sort of shrug your shoulders and be like, ‘Okay, whatever, I’ll make up for it when I’m 40.’”

and:

“Being super busy and prioritizing your grades and classes over your health could also have an effect too. If you get home and you don’t feel like cooking something healthy, it’s really easy to just have something that’s really not good for you. And I think that can lead to a decline in your mental health and your physical health and ultimately it will probably just hurt you more in the end.”

A lack of a stove and/or fridge limited preparation ability and influenced the types of foods purchased by students. Not being able to cook meals subsequently led to a limited control over what and how to eat and further perpetuated food-based decisions of convenience rather than nutrition content. Students expressed:

“I kind of make food choices off of convenience rather than actual healthy food or something that I want to eat. I don’t have a stove, so I put mozzarella sticks in the microwave for a couple minutes and done. But it’s more of a convenience.”

and:

“I think [not] having a kitchen is a very big part of it for me just now because I don’t have as much control over the food I eat. I feel like I tend to make a lot worse choices.”

Students also reported changes to their dietary choices and food behaviors due to barriers to healthy eating such as limited resources and the cost of healthy food.

“I eat cheaper food now. I need to find ways to make things last longer so I get cheese that’s wrapped in packages and stuff like that.”

and:

“Vegetables tend to be more expensive than meat. So, you wouldn’t buy the vegetables because when you’re a college student, you want to save as much money as you can.”

With regard to ‘awareness of food marketing on dietary behavior’, students described being aware of a variety of diet trends including keto, paleo, vegan and intermittent fasting and how diet trends influenced eating environments at greater contexts. As one student said:

“It’s the same with fad diets. [...] Chipotle, you can walk in and order like specific things like a keto diet bowl and they’ll have set ingredients that go into it, or paleo, or vegan or anything based off of a diet. As fad diets are becoming more popular, they try to sell you on them.”

Social media was identified as a platform that exposed students to diet trends, which in a few cases resulted in students making changes to their own eating behaviors. Discerning quality and context of information was also necessary to be able to utilize information. As two students stated:

“The market is so flooded that when you have the super diet websites that are putting out crazy untested ridiculous [...] they will be like ‘oh you should have a fruit smoothie every morning or like 3 times a day’. It’s still not balanced and doesn’t make any sense.”

and:

“They’re coming out with trend diets that are high fat diets and you can’t eat high fat and high carbs, so like, you have to be aware of what you’re eating.”

#### 3.2.5. Critical Nutrition Literacy

With regard to ‘critical appraisal of nutrition information’, students were aware of a variety of sources from which they could obtain information on nutrition, food production and overall health and wellbeing. Students associated the credibility of source with the institution status (e.g., government agency, research institution) and primarily used the internet and social media to seek scientific studies, medical professionals and government websites for credible information related to food, health, and nutrition.

Other modalities for obtaining information included written (books), audio and visual content. Students also reported it was necessary to engage in strategies to assess information accessed online and other media as misinformation and bias is a common concern. One student stated:

“You can watch documentaries that are against stuff and it’s so biased because that’s what they want you to think, it’s bad to eat something.”

Research and research studies were the comparative standard and students used strategies such as a “triangulation” of sources as a way to confirm information. While ideal, to a few this was also viewed as burdensome for the consumer. Additionally, the amount of information available and accessible and the effort required by the consumer to confirm information could inadvertently lead people to be less critical of information presented. As one student described:

“You can find thousands of different results on the internet for something with the same question. So, the only way to really know what you’re going to be looking at is to kind of do an analysis of all the research that you’re seeing and cross reference it and look at how they did it. […] Either way that takes a lot of time and energy. And I feel like for a lot of people, they just kind of look at whatever’s going on TV and are like, ‘Wow, that sounds great!’”

Students also seemed to inherently trust internet information. While students recognized the potential of misinformation and credibility issues with information accessed online, the internet was a primary tool to access credible and reliable information related to food, nutrition, and health. Specifically, students reported relying heavily on the search engine Google to search for information related to nutrition and health including seeking out a health professional. One student stated:

“I would Google anything just to find out what the right thing to do is [...] or at least figure out if I should go see a nutritionist.”

Understanding that there could be a plethora of results for any topic, students relied on the assumption that results from any search were organized by level of credibility. Top listed results were typically viewed as the most credible source of information. As one student shared:

“I’m just hopeful. I go based off of what comes up first. And if it’s at the top of the results I’m kind of just going to assume that’s more credible.”

With regard to ‘awareness of societal barriers to good health’, students brought up topics that focused on overall health and the importance of marketing healthy behaviors in general on the college campus. Peers’ influence on health behaviors and “welcoming feelings” to go to places such as the recreation centers and dining halls on campus were common. Students stated, “Yeah, gyms are a social nightmare” and, “Yeah I wish I could go to the gym more. I would love to go exercise more often, but I’m just like so scared that people are going to judge me and that kind of motivates me to [say], ‘Yeah, let’s change that.’ You know?”. Solutions to overcome societal barriers to good health were brought up in the focus group in terms of feeling welcome to exercise at the campus recreation center:

“I wonder if there’s something we could do, like a gym buddy system we could just set up as students. You know? Like, I don’t know, make some sort of an online sign-up sheet where people go to the gym in pairs? Like, I’d love to do something like that.”

and addressing the food at the dining halls:

“People listening! You would have to get a lot of people to just like not pay for a dining plan and then they would be like, ‘why are we losing money, like why aren’t people paying for a dining plan?’”

However, when considering following through with making healthful changes on the college campus there were issues with individuals’ feeling of power, being successful with implementing changes, and how to go about advocating for change:

“I don’t think the group collective is strong enough right now. Everyone has their own individual thoughts but we haven’t collectively put them together and organized them.”

“I’ve been wondering about it because yeah, how could this information—how could everybody get this information out there. The majority of processed foods are really awful for us and how does this information get out, I’m not sure, but it seems like nutrition is the leading cause of all mental and physical health problems in America. There’s more studies coming out about it, how do we get people to stop ignoring what we know? Not just with food, but in all sorts of things. I don’t know.”

In summary, nutrition literacy was broadly described by this sample as a knowledge or awareness of food and nutrition-related terms and the impacts of food and eating behaviors on the physical body. A variety of factors influenced food-related decisions and eating behaviors of college students and students sought credible information primarily online. Although students engaged in strategies to discern credible sources for information, they also relied on intuition and informed assumptions to make food-related decisions.

## 4. Discussion

The purpose of this study was to determine how nutrition literacy domains (functional, interactive, critical) influence the dietary decisions of college students. This study found that the three domains of nutrition literacy played a role in college students’ food choices and how they navigated their food environment. Many food-based decisions by college students occurred in the functional domain of nutrition literacy, i.e., food-based decisions made from basic, factual health information. Alternatively, the application of food and nutrition-related information gained from a variety of media (e.g., social media) might be challenging for college students due to circumstances unique to the college environment. Students might also inadvertently be exposed to misinformation due to a lack of ability to identify credible sources when seeking information on the internet and need support to advocate for more healthful environments on their college campus.

The findings from the current study built upon the importance of understanding and assessing nutrition literacy. Nutrition literacy has been established as an important construct related to dietary behaviors in parents of young children in which parents with higher nutrition literacy were more likely to have children with BMI-z scores within a healthy range [11]. A positive relationship has also been established with nutrition literacy and people with chronic disease in that there is a better adherence to dietary prescriptions in people with higher nutrition literacy scores [18]. However, less is known about the role nutrition literacy plays in college students’ food choices. Understanding the impact nutrition literacy can have on eating behavior is especially important in this population because college students are at a unique time point in their life where many of their dietary behaviors are influenced by peers and their food selection choices are limited due to commonly reported barriers with food access and availability [19,20].

As Velardo and Drummond [21] previously demonstrated in focus groups with youth populations, the findings in this study reflected the constructs of the SEM. College students referred to individual, interpersonal, organizational and community factors when discussing the different nutrition literacy domains. For example, in terms of individual factors, the majority of responses and definitions of nutrition literacy were in the context of factual knowledge or the knowledge of basic facts (e.g., what MyPlate is), which was the basis of nutrition literacy as opposed to procedural or facilitating knowledge [22,23]. Interpersonal and organizational constructs of the SEM were identified within the interactive nutrition literacy domain. Within this domain, students described more procedural knowledge or decision-making skills such as navigating the college food environment and awareness of the role food marketing has on eating behaviors. The community construct from the SEM was addressed in the critical nutrition literacy domain. Within this domain, facilitating knowledge or the combination of factual knowledge, procedural knowledge and decision-making skills together, was described by students when critically appraising nutrition information and reflecting on societal barriers to good health.

As college students discussed the questions that were designed to reference functional nutrition literacy, much of the conversation focused on (1) “just knowing” a food was healthy and (2) that many people use calories to determine the healthfulness of food items. The idea of “just knowing” that a food was healthy might be indicative of a perceived strong foundational knowledge of basic nutrition information. However, as most of the participants focused mostly on the calorie content of food and limiting carbohydrates, it was evident that their perceptions might not reflect the importance of variety and a well-balanced diet to achieve the best health outcomes [24].

Research by Werner and Betz [25] showed similar findings in that the majority of their college student sample (*n* = 71) was aware that dietary recommendations existed but less than 25% could correctly identify any one recommendation. Furthermore, the dietary behavior of the current study’s sample was poor with the majority of people not meeting the DGA recommendations for fruit and vegetable intake. This brings into question how students can successfully/healthfully navigate their food environment while also having possible misperceptions about healthful eating behaviors.

During the discussion of interactive nutrition literacy, students expressed difficulties with navigating their food environment due to friends influencing unhealthful eating behaviors, drinking alcohol, staying up late studying and having limited access/availability to a variety of appetizing and healthful foods. Similarly, the factors of peer influence and healthful eating environments also emerged during the development of the Behavior Environment Perception Survey (BEPS), which was designed to assess how college students perceived their college environment [26]. During the survey validation, these two factors (peer influence and healthful eating environment) were found to predict fruit and vegetable intake, sugar sweetened beverage intake and how many days/months young adults felt healthy and full of energy [26]. These results underscore the importance of understanding the role of interactive nutrition literacy on young adults’ eating behaviors and health outcomes.

College students were also aware of the promotion of different eating trends in their food environments and discussed how their eating behaviors had changed or how they had questioned their eating behaviors as a result. For example, one student shared that they learned about veganism through social media and then changed their eating behavior to become vegan. This was a prime example of a college student using information that they had gathered through social media and making a procedural change that might affect their health positively or negatively if they were not meeting their full nutritional needs (i.e., B12 intake). Other research has shown both positive and negative relationships with social media engagement on eating choices. Walker et al. [27] surveyed college women (*n* = 169) on their use of Facebook and factors related to disordered eating. Those who used Facebook more and also engaged in greater physical appearance comparison were more likely to report disordered eating behaviors, but their results also showed that women who did not engage in physical appearance comparison were less likely to report disordered eating behaviors. There is much to uncover about the role of social media on eating behaviors and how young adults are using this abundance of information to make health decisions.

Interestingly, during the conversation around critical nutrition literacy, college students expressed the sentiment of experiencing information overload but perceived their own skills in understanding reliable nutrition information to be high. Based on the focus group discussion, the most frequently utilized and “trusted” online sources by college students for seeking out nutrition information were Google and social media. Students reported that they “just know” which sources of information are credible. Other research supports that young adults are looking to social media (Facebook, Twitter, YouTube) to gather health information, especially when facing specific health conditions such as diabetes or a common mental health disorder [28]. However, future research is needed to understand the efficacy of sharing reliable health information through these platforms and how to assess young adults’ critical nutrition literacy skills [29].

Instruments have been validated such as the Revised Critical Nutrition Literacy Tool (CNLT-R) [30] and the critical nutrition literacy-evaluation (CNL-E) scale [31] to assess young adults’ critical appraisal skills of media and evidence-based claims of nutrition information. However, these instruments do not assess the perceived reliability of other common sources of information that college students are using such as social media platforms and do not assess the advocacy component of critical nutrition literacy.

The other component of critical nutrition literacy as described by Velardo [13] is the idea of social engagement/advocacy for a more healthful eating environment for all. Throughout the conversations, students expressed negative viewpoints about their eating environments and the limited availability of food that was both appetizing and healthy. However, when asked “What would motivate or motivates you to change your environment to make it more healthy?”, participants expressed more broad examples of societal barriers to health referencing the social environment of the campus (e.g., Greek Life) and challenges with making substantial changes such as the need for financial investments and buy-in from large groups. Research has demonstrated that young adults play an important role as a catalyst of change for a more healthful environment [32,33]. The themes that emerged within this domain highlight the importance of supporting students in their ideas and to encourage social advocacy in young adults in the hope that this motivation to create more healthful environments will continue into adulthood and older adulthood.

This study was not without limitations. The participants self-selected to participate in the focus groups and while advertisements were distributed campus-wide, the people who volunteered to participate may have had a special interest in discussing their eating behaviors. Additionally, this study was conducted on one college campus with a primarily homogenous sample in the Northeast and may not be representative of other four-year institutions. The strengths of this study include an in-depth look into how young adults make eating decisions while navigating their college food environment and how the different factors of nutrition literacy play a role in their decision-making process.

## 5. Conclusions

Nutrition literacy has been established as an important indicator of healthful eating in adults and children with higher nutrition literacy scores being associated with a more healthful diet. The findings from this study suggested that nutrition literacy also played a role in college students’ eating behaviors and how they navigated their college food environment. Our findings supported that all three domains of nutrition literacy influenced young adults’ eating decisions. This indicated that nutrition literacy as a whole is an important construct to measure when aiming to influence young adults’ eating behaviors. Given the qualitative nature of this study, future research should aim to quantitively assess young adults’ nutrition literacy and examine the relationship between nutrition literacy and diet quality. By better understanding how the multidimensional construct of nutrition literacy relates to eating behaviors, programs can be developed on college campuses that are relevant and effective at encouraging more healthful behaviors in students.

## Figures and Tables

**Table 1:** Emergent themes and additional supporting quotes organized by nutrition literacy domains.

Domain	Themes	Supporting Quotes
**Functional**	**Food enhances or inhibits good health**	“How I feel after I eat something, it kind of lets me know how healthy I would say it is, right? Because if I feel really gross afterwards then it probably wasn’t that good for me then to put in my body”. “There’s a lot of food that I just know is healthy, fruits and vegetables, lean meats and fish” and “oh, salad; it’s healthy”. “Healthier food you can just kind of taste [...] I don’t know, there’s like a certain freshness or like lightness to things I would consider healthy. Even a healthier meat, I feel like tastes healthy”. “I figure generally if something is grown from the earth, then it’s probably pretty healthy compared to anything that comes in a box”.
	**Components of a healthy diet**	“Just how many calories are in the amount of foods I eat. I don’t really look at carbs, or sugars, or nutrition labels. Just information like, Oh like, a hundred calories, yay, okay”. “I think calories in versus calories out is also a very important one. Just because while it’s not necessarily a good way to monitor nutrition, usually if I set a calorie limit for the day I’m not going to blow it all on a few cookies. I’ll actually eat vegetables and things that are lower calorie and healthier for you in order to meet that and also be more focused on exercise so that I can eat more food”.
**Interactive**	**Navigating the college food environment**	“It’s easier to eat like garbage when I am eating with my family or friends. If we’re all going out for drinks or something and I have a couple drinks I want tater tots or something that is garbage. Versus if I’m just cooking for myself and just eating by myself then I’ll just cook sweet potato and green beans or something for dinner and be completely satisfied and happy with that”. “I think if you add in drinking cultures and now marijuana smoking, that definitely affects people’s diets”. “I feel pressured to go out and eat food at restaurants more because I know a bunch of my friends will go out”. “It’s hard to keep vegetables in a dorm room”. “Usually when you get home you’re burned out from the entire day. Some people have 8:00 a.m.’s and have really late labs. So you tell yourself you’re going to cook in the day, but then you get home and it’s like, ahhh, just eat, like, McDonalds or something”. “Eating sporadically, I could get really drunk on a Friday night and eat a huge meal at like 2AM, [...] or go a day without eating just because I’m studying or busy just like didn’t even think about it”.
	**Awareness of food marketing on dietary behavior**	“There are a lot of diets [...] the keto diet, the paleo diet, intermittent fasting, that have gotten a lot of attraction because they are just present on social media”. “I’m actually vegan, so I feel social media probably had a pretty big impact in me making that decision. Mostly just becoming aware of it I guess and then doing my own research, YouTube, the meat industry”. “I look up a bunch of stuff about veganism because it intrigues me. [...] I do see YouTube videos [...] of people’s journey with it and how it affects them”. “The market of it is so flooded that when you have the super diet websites that are putting out crazy untested ridiculous [...] they will be like ‘oh you should have a fruit smoothie every morning or like three times a day’. It’s still not balanced and doesn’t make any sense”.
**Critical**	**Critical appraisal of nutrition information**	“Maybe a government website, a health sponsored, like green initiative. A government website I would probably trust more. Like .gov”. “I personally, three years ago found a YouTube channel called nutritionfacts.org and I love that channel. This doctor, Dr. Michael just goes through just a lot of nutrition studies and all he does is just state what they find in the studies, it’s just no opinions, just what they found in the studies. That’s where I get the bulk of my nutritional knowledge”. “I typically go to a book or something. Usually it’s a textbook, I look up the basic biochemical stuff. And a lot of the stuff you find online isn’t accurate”. “Specialized institutions within those fields. If the National Heart Association said something, I would probably be more likely to believe them”. “It’s really hard, it’s like you have to open up a book and read it [...] and look at actual studies and that’s really the only way you can truly get nutritional advice and guidance. I try to look at scholarly articles and look for the raw data, because anyone can say anything it’s the internet”. “I tend to try and triangulate my sources. If I can find the same piece of information repeated from three relatively reputable sources, it’s probably good”. “I’ve also done my own research and usually just go to the internet for that”. “Google, I google everything”. “I definitely Google everything. It sounds horrible but it’s the quickest and easiest baseline result. I still start at my phone and then call my mom and ask her if I should go to the doctor”. “I usually trust the first thing that pops up. There’s the fact box with the information you need and then there’s the websites. I don’t trust the websites and I never click on them, I just look at the box”.
	**Awareness of societal barriers to good health**	“The Greek community put handprints on a big anti-hazing banner and I felt like that was a cool change where everyone came together”. “If it were easier to do, like, I feel like you can say that you want changes and even get a big group of people together to do it but it’s a big campus and I don’t think it would be that easy to do and considering a lot of the problems like fresh produce and stuff like that, that’s expensive and it goes bad quickly so like I understand that it isn’t always feasible”. “…knowing that I have a lot of family members in the area that are probably going to come through this system as well, that kind of helps a little bit with the motivation and I’m not really sure where that could even start”.

## Data Availability

The data presented in this study are available on request from the corresponding author. The data are not publicly available due to privacy restrictions.
